# Peripheral Neuropathies Seen by Ultrasound: A Literature Analysis through Lexical Evaluation, Geographical Assessment and Graph Theory

**DOI:** 10.3390/brainsci11010113

**Published:** 2021-01-16

**Authors:** Daniele Coraci, Claudia Loreti, Augusto Fusco, Silvia Giovannini, Luca Padua

**Affiliations:** 1 UOC Neuroriabilitazione ad Alta Intensità, Fondazione Policlinico Universitario A. Gemelli IRCCS, 00168 Rome, Italy; danielecoraci@aol.com (D.C.); claudia.loreti@policlinicogemelli.it (C.L.); augusto.fusco@policlinicogemelli.it (A.F.); 2 Department of Neurosciences, Università Cattolica del Sacro Cuore, 00168 Rome, Italy; 3 UOC Riabilitazione e Medicina Fisica, Fondazione Policlinico Universitario A. Gemelli IRCCS, 00168 Rome, Italy; silvia_giovannini@yahoo.it; 4 Department of Orthopaedics and Geriatrics, Università Cattolica del Sacro Cuore, 00168 Rome, Italy

**Keywords:** ultrasound, peripheral neuropathy, anatomy, personalized medicine

## Abstract

(1) Background: Ultrasound is a well-known tool used for the diagnosis and management of many diseases, including peripheral neuropathies. The main aim of this study was the lexical analysis of the literature on this topic considering the most cited words and the relationship between the words and the papers. Furthermore, a geographical analysis was performed to evaluate the worldwide prevalence. (2) Methods: We performed a literature search on PubMed, and we calculated the occurrence of the words indicating nerves and the body parts. Furthermore, we calculated the number of papers for each country, considering the affiliation of the first author. Finally, to describe the relationships between the words and the papers, we used the 30 most cited words, and we built a matrix describing in which papers a word was cited. This matrix was used to create a network based on the graph theory using Gephi 0.9.2 software. (3) Results: The most cited nerves were median and ulnar ones, and the most cited body parts were hand, wrist and elbow. The United States of America was the most productive country, with 80 papers. The graph of the network showed the importance of ultrasound as support for therapy. (4) Conclusions: The study represents a lexical analysis of the literature and shows information about subjects, authors and relationships of the papers. This may be helpful for better understanding and evaluation of the situation of the current literature.

## 1. Introduction

The application of ultrasound (US) in medicine is well known and relatively old. The first research papers about this technique for diagnostic purposes were published in the 1960s [1,2]. At present, US is a fundamental tool for the evaluation of patients affected by different diseases [3,4,5,6,7,8]. US has proven to be particularly useful for the study and diagnosis of peripheral neuropathy [9,10]. It is able to evaluate the morphological features of the nerves and their changes in several conditions [6]. In particular, the application of US in nerve assessment provides some information not obtainable by the clinical and neurophysiological examinations alone [11,12,13]. 

US provides a tool to image and see the organs and can clearly measure and locate the nerve damage [14]. This is fundamental because it allows the understanding of the severity of the pathological condition. US can be used to distinguish between axonotmesis and neurotmesis, with essential consequences for the therapeutic decision [15]. Furthermore, US can directly evaluate intrinsic (e.g., anatomical variants) and extrinsic abnormalities (e.g., compression by surrounding structures) [16]. Additionally, even muscle evaluation through US delivers informative data about a nerve lesion [17,18]. For example, the depiction of a minimal muscle contraction can let us suppose a partial saving of nerve fibers. Thanks to these characteristics, US should be considered a valuable research and clinical tool, and it should be implicated in clinical practice and in neurophysiological labs. Indeed, the strength of US is palpable when associated with neurophysiological evaluation, which can, instead, quantify the functional damage. Of course, both the approaches (US and neurophysiology) have to be preceded by clinical assessment, which represents the basis for the diagnostic suspicion [19].

Besides diagnosis, US shows effectiveness for the whole management of neuropathy, particularly important for more fragile patients frequently presenting multimorbidity and polipharmacology [20,21,22,23,24]. As previously mentioned, the correct damage classification implies a correct treatment decision (conservative or surgical) [25,26,27]. Additionally, US is essential during mini-invasive treatments [28]. The US-guided injection of specific drugs for anesthesia and pain relief is a routine approach that takes advantage of US abilities [14,29,30,31]. Seeing the nerve and the needle during the procedure allows an incredible increase of the precision, drastically reducing the risk of intravascular dissemination of the drug and the other possible side effects [32].

The literature on US application in peripheral neuropathies has increased during recent decades, with robust evidence. A large number of scientific papers support clinical activity, providing evidence-based guidelines for diagnosis and treatment. Despite the growth in published studies, how US is used in clinical and research practice remains poorly understood [33]. Furthermore, we should always keep in mind that the literature can be read from different points of view, and its exploration can reveal interesting elements that can be used for reflection. For example, the geographic information, based on first author affiliations, can reveal the impact of a subject in different countries, or the time distribution may allow the development of interest in a specific matter. However, the most powerful information is probably obtainable by lexical analysis. Studying how words are used to convey concepts may elucidate some facets of the scientific knowledge about a matter. In this work, we used different approaches to review the literature on diagnostic US in peripheral nerve diseases. 

The aim of our study was the evaluation of the present literature on nerve US through the following points of view: (1) the most studied nerves and body parts; (2) the geographical origin of the authors; (3) lexical analysis based on the most used words in the abstracts. With the application of the listed approaches, we aimed to analyze the literature in order to provide information useful for the comprehension of the literature evidence. In particular, with the lexical analysis based on this novel approach, we intended to show which words are mainly used in scientific literature on nerve US and how the same words are connected with the papers.

## 2. Materials and Methods

The literature search was performed on PubMed using the following Medical Subject Headings (MeSH) terms: “peripheral neuropathies”, “ultrasound” and “diagnosis”, associated with the Boolean operator “AND”. The following filters were used: article type (meta-analysis, systematic reviews, reviews, randomized controlled trial and clinical trial), language (English) and publication date (the last 10 years). The complete results, including the abstracts, were exported as a text file using the PubMed function. This file was imported into the freeware software TXM 0.8.0 (Copyright © 2010–2018 ENS de Lyon, University of Franche-Comté, CNRS), able to count the frequency of a word in texts (in our case the titles and the abstracts of the papers) [34]. In particular, we calculated the total occurrence of nerves usually assessable by US and body parts (for example, head, arm, foot) mentioned in the titles and abstracts. These calculations were expressed as percentages of the total words counted for each category (nerves or body parts). Furthermore, we collected data about the geographical origin of each paper, using the country in the affiliation of the first author. Finally, in order to perform the lexical analysis, we applied the lexical network based on graph theory (LENGTH), a method able to describe the relationships between the papers and a list of words present in their titles and abstracts [35,36]. To build the graph, we selected the 30 most cited words in the file exported from PubMed, considering the words consistent with the research topic. This selection was performed with the support of the abovementioned TXM 0.8.0 software. Hence, we calculated the occurrence of these words in the title and the abstracts of the papers, obtaining a matrix with values “1” or “0”. The value “1” indicated the presence of a specific word in a paper and “0” its absence. The created matrix was imported into the freeware software Gephi 0.9.2 (licensed under CDDL and GNU GPL 3) to build the relative graph [37]. This is a network where the nodes represent the words and the papers, while the edges represent the connections between them. Additionally, the software allows the calculation of measures describing the centrality and the impact of the single nodes. In particular, the degree indicates the total number of connections from a node, that is the frequency of the word. The closeness centrality and the betweenness centrality are respectively related to the length of the paths between the nodes and to the presence of a node between two others. They refer to the importance of the nodes inside the graph. In our model, these two measurements of centrality are generally related and may be linked to the diffusion of the words in titles and abstracts and to the strengthening of the connections of the word and the papers. 

## 3. Results

**Figure 1:**
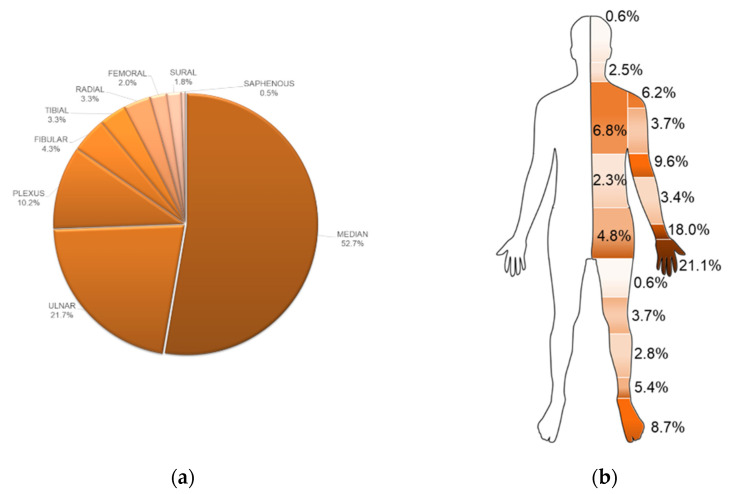
(**a**) Percentages of the citations of the most common nerves assessed by ultrasound (US). (**b**) Percentages of the citations of the body parts.

The total number of papers on PubMed, found with the described search, was 282 (on 1 November 2020). The absolute number of publications, visible in the graph visualizing the results per year, was growing with a peak in 2018. The analysis of the words revealed the clear dominance of the median nerve (52.7% of the cases), followed by the ulnar nerve (21.7%) and the plexus (10.2%). The other nerves were present with very low percentages (<5.0%) (Figure 1a). Among the papers, 27 presented multiple nerves, and one assessed all the common nerves but the saphenous. Considering the association of nerve couples, 15 papers simultaneously evaluated median and ulnar nerves, 7 papers median and radial nerves, 8 papers ulnar and radial nerves and 6 papers fibular and tibial nerves. The two papers evaluating the saphenous nerve also studied fibular, tibial and sural nerves. Finally, among the uncommon nerves scanned with US in clinical practice, the vagus nerve was present in one paper, the obturator nerve in two papers and the pudendal nerve in three papers. The musculocutaneous nerve, although relatively frequent in US practice, was just present in one single work. The most frequent body parts referenced in the papers were the hand (21.1%) and the wrist (18.0%) followed by the elbow (9.6%). The most common part of the lower limb was the foot (8.7%), while the head and the thigh were the least named (0.6%) (Figure 1b).

**Figure 2:**
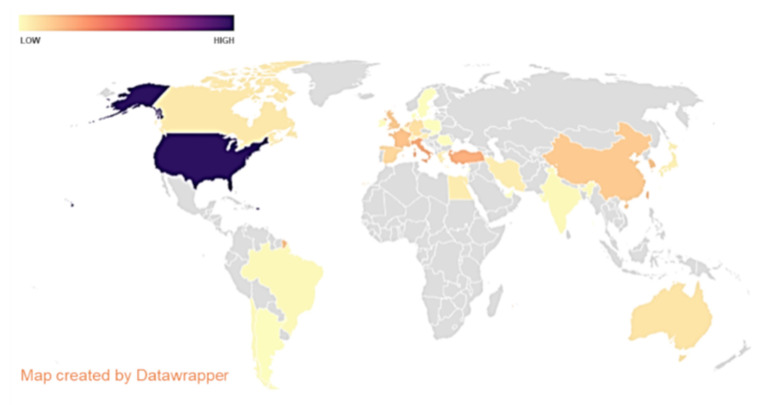
Literature production of the countries. Gray represents the countries with no associated papers.

Considering the geographical origin of the literature production, the United States of America (USA) was the most productive country (28.4% of the papers), followed by Italy and Turkey (7.1% and 6.7%, respectively). However, with this search, every continent showed its own contribution to the literature production, albeit with dissimilar impact. Emblematically, the Arabic Republic of Egypt was the unique African State, while Europe showed not only a large number of countries but also a large number of papers (Figure 2).

**Figure 3:**
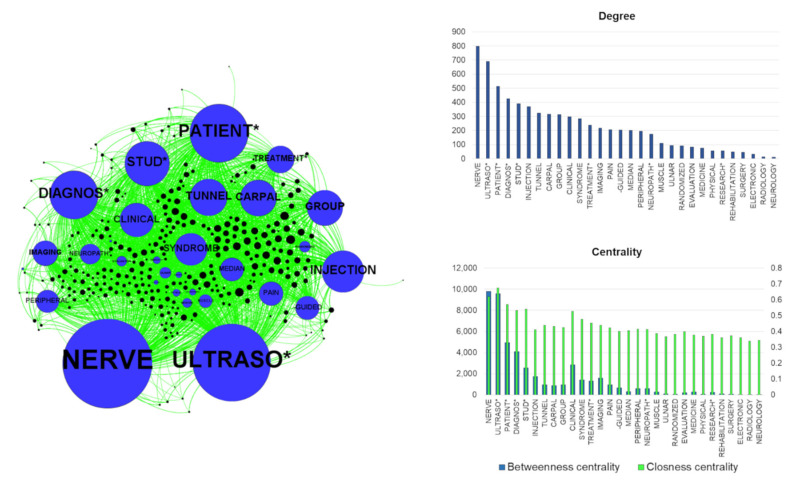
On the left, the graph of the LENGTH method. The nodes (the circles) represent the words (blue) and the papers (black). The dimension of each node is related to the degree values, meaning the number of connections with papers and, hence, the frequency of citations of the words in the titles/abstracts. The edges (green) represent the connections between a word and the papers. The node distribution is based on the ForceAtlas2 algorithm. This algorithm distributes the nodes depending on their connections; closer nodes are more connected. On the right part of the figure, the histograms indicate the values of degree, betweenness and closeness centrality of the word nodes. The asterisks represent different final parts of words (for example, ultraso* may indicate ultrasound, ultrasonography, etc.).

Considering the LENGTH approach, the graph of the network showed some peculiarities. In particular, the whole network was separable into two main parts: on the left side, the topics about the diagnosis were visible (main words: DIAGNOS*, IMAGING, CLINICAL, EVALUATION), while on the right the topics about the treatment could be seen (main words: TREATMENT*, INJECTION, -GUIDED) (Figure 3) [38]. In the latter part were located the elements associated with the US support for injection and some specific symptoms, especially pain. Additionally, in this right part of the graph, the words about carpal tunnel syndrome were present. Among the 30 selected words, NERVE and ULTRASO* (the asterisk indicates different possible parts of words: ULTRASOUND, ULTRASONOGRAPHY, etc.) presented the highest degrees, hence the major connections with the papers. These two words had the highest centrality values, confirming their importance in the network. Among the most frequent words appeared CARPAL and TUNNEL, the only words directly indicating a specific disease. For these two words, the degree was relatively high, but the centrality values were lower. Furthermore, the only two nerves appearing in this 30-word list were MEDIAN and ULNAR. The first of these showed the highest degree and centrality values. Finally, most of the papers with the highest degree, meaning a large number of connections, were located in the right part of the graph, where we can find treatment topics.

## 4. Discussion

Our study shows that the current scientific research on nerve US is largely focused on upper limb nerves and is mainly produced by a few countries, although from every continent and independent of the country population. Furthermore, the lexical network, presented with the LENGTH approach, shows the most frequent words in the papers (mainly belonging to diagnostic and therapeutic semantic fields).

The information obtained by the described approaches illustrates the literature analysis from novel points of view. They reveal some features of the literature useful for speculation. Furthermore, they can disclose some messages conveyed by the words used in the published studies. Based on our results, we can highlight how the most assessed peripheral nerve structures by US are median and ulnar nerves. This consideration is supported by the evaluation of the most treated single body parts: hand, wrist and elbow. This is expectable, especially because of the relative ease in accessing these nerves and the common diseases involving these structures [25,39,40,41,42,43]. The other nerves are not visible in the graph because of their very low frequency in titles and abstracts of the papers. Presumably, these other nerves are not as commonly studied with US, which is why they are less reported in the literature. This issue should stimulate research activity to increase the studies about other anatomical sites and nerves. Interestingly, as visible in Figure 3, carpal tunnel syndrome, which affects the median nerve and represents the most common peripheral nerve entrapment [25], is associated with US even from a therapeutic point of view [44]. Obviously, the graph considers very relevant, for degree and centrality, the words directly related to the search. However, some other data are obtainable by the comparison of the different graph parameters. If diagnosis is one of the main topics used for the research, the centrality of the treatment is relatively high, and this can provide some stimuli for the analysis. Indeed, the US-guided injection of drugs for inflammation and pain treatment is well known, as is the US-guided administration of botulinum toxin for the treatment of spasticity and dystonia [45,46]. Hence, the literature shows how US is fundamental for complete patient management, for diagnosis and for therapy. This is also supported by the visible size of the nodes representing the single papers close to the treatment. These nodes are very large, and this indicates their high degree and, hence, the high number of connections of these papers with the words. This may mean the papers related to the treatment are related to many other subtopics (like carpal tunnel syndrome, pain or injection). Notably, the surgical approach is rare in our literature analysis, and this probably highlights the preference for conservative treatment, despite being a discussed topic [47,48]. In our research, limited space is observable for rehabilitation. Of course, it can depend on the search strategy (see MeSH term “diagnosis”), focused on the diagnostic contribution of US to peripheral nerve study. However, besides the words used for the research, the impact of the therapy (and not only of the diagnosis) is certain. Indeed, the node that referred to therapy with the highest degree (the major frequency) is INJECTION with a degree similar to DIAGNOSIS. This may reveal the importance of US in the management of the peripheral nervous system for diagnosis and therapy based on drug inoculation. The word TREATMENT*, although less common than INJECTION and other words, presents high importance (shown by a relatively high level of centrality). This may mean that the association of US and treatment is relatively spread in the selected papers. The values of degree and centrality are low for rehabilitation [16,49]. This can be used to reflect on the importance of rehabilitation of the peripheral nerve diseases in the literature [50]. Additionally, these data let us speculate about the possible limited use of US in rehabilitation. 

Finally, the geographic analysis of the literature shows the very widespread interest in the matter all over the world. There are different levels of literature production independent of the population. The high number of papers (80) published by the USA confirms the high level of production from this country. However, the USA generally represents a very prolific country in literature production, and further analysis should be conducted to better understand the real meaning of this point. Further speculation is possible from the other data. Notably, in our study, the two other productive countries are Italy and Turkey. Indeed, this demonstrates a growing interest in nerve US for these two countries in recent years. Interestingly, two other very populated countries (China and especially India) do not present a comparable level of literature production, and this may be related to their relatively recent interest in the topic analyzed in this study.

The study obviously shows some limitations. First, the search was performed on PubMed using specific filters (article types, date and language). This probably caused a simplification of the data collection with a possible lack of some literature studies. However, we chose these filters in order to analyze the recent studies with the highest evidence values. Another limitation is linked to the method itself, which was developed to assess the words in the abstracts and not in the whole paper. This can sound like an important restriction for the analysis. However, the abstracts can be used everywhere without the necessity to download the whole papers, and this allows a general lexical evaluation of the considered studies. Finally, the choice of the words is related to researchers’ experience and should be properly adapted (in number and type) for each lexical analysis.

## 5. Conclusions

In conclusion, the presented approaches shed light on some aspects of the literature. The study is based on a lexical analysis of the literature and may be useful for the comprehension of the lexicon used for the definition of specific topics. The data support reflections about subjects, authors and relationships of the papers. With our analysis, we found that evidence of US application for the diagnosis and treatment of peripheral nerve diseases is large. However, there is a certain unbalance considering the studied body segments; upper limbs, median nerve and ulnar nerve are the most studied, while the nerves in the lower limbs are relatively rare in the literature. Additionally, the geographical assessment reveals a large diffusion of the literature on nerve US all over the world but with important inhomogeneity among the countries, independent of the population. Finally, the LENGTH approach displays the relationships between the words and the papers, showing the importance of US for diagnosis in clinical practice and for the guiding of treatment, especially in carpal tunnel syndrome. With the proposed deep analysis, we can see the potential and limitations of the current literature in a new way. We hope this approach can support the definitions of new research studies for better use in clinical practice.
